# Influence of environmental factors on macrofoulant assemblages on moored buoys in the eastern Arabian Sea

**DOI:** 10.1371/journal.pone.0223560

**Published:** 2020-01-30

**Authors:** M. V. Martin, Ramasamy Venkatesan, M. Beyline, V. P. Limna Mol, L. Divya

**Affiliations:** 1 Ocean Observation Systems, National Institute of Ocean Technology, Chennai, Tamil Nadu, India; 2 Department of Zoology, Central University of Kerala, Kasaragod, Kerala, India; 3 School of Ocean Science and Technology, Kerala University of Fisheries and Ocean Studies, Kerala, India; CIIMAR Interdisciplinary Centre of Marine and Environmental Research of the University of Porto, PORTUGAL

## Abstract

Factors governing the distribution of organisms in the pelagic ocean are understudied. In this paper we describe environmental parameters and macrofouling assemblages on 11 buoys deployed in the Arabian Sea for an average duration of 322 days. Macrofoulants on all the mooring components extending from the sea-surface to a depth of 1800–4300 m were documented. Role of temperature, salinity, dissolved oxygen, biological productivity and zooplankton community in governing the macrofoulant distribution are described. Species composition, vertical zonation and wet biomass exhibited significant spatial variations. *Lepas anatifera* constituted more than 90% of foulant wet biomass on all moorings. Assemblages in the southeastern (SEAS), east-central (ECAS) and northeast (NEAS) regions were distinct. Density of *L*. *anatifera* on surface buoys were low in SEAS (0.2±0.09 no./cm^2^), high in ECAS (0.32±0.11 no./cm^2^) and moderate in NEAS (0.23±0.04no./cm^2^). Macrofoulants were observed up to a depth of 75 m in SEAS, 130 m in ECAS and 120 m in NEAS. The depth profile of macrofoulant assemblages on moorings could be related to the prevalent hypoxic condition. Vertical profiles of wet biomass on all moorings exhibited subsurface maxima at depth ranging from 10 to 20 m, consequent to the abundance of *L*. *anatifera* in a thermally stable depth of water column, wherein diurnal and semidiurnal temperature variability was minimal. We attribute the observed variation in fouling assemblages to dissolved oxygen levels, salinity and diurnal variability in temperature and salinity.

## Introduction

Gaining insight into factors that govern patterns of assemblages is a fundamental objective in ecology. Assemblages are shaped by biotic and abiotic stresses in their respective environments. Mobile and sessile organisms respond differently to the stresses. While the mobile organisms could survive a stressful condition by retreating to a favorable environment, the survival of a sessile organism is determined by tolerance to stresses[1]. The study of sessile assemblages may provide information on the habitat and health of the ecosystem. When compared to terrestrial assemblages, sessile assemblages in marine environments are relatively inaccessible and understudied[2]. Of marine studies, most are of intertidal, subtidal and benthic habitats[3–9]while sessile assemblages in the pelagic ocean are rarely studied.

Pelagic ecosystems are highly dynamic and are shaped by multiple interrelated scales of biophysical interactions [2]. Crucial environmental variables such as temperature, salinity, light, pH, dissolved oxygen (DO), nutrients, etc. show a strong vertical gradient in the pelagic ocean. Man-made stationary structures in the ocean accumulate sessile assemblages across the vertical gradients in environmental parameters.Assemblages of sessile organisms exhibited notable regional variations and vertical zonation on wind farms [10,11], petroleum platforms [12–15] moored buoys [16–18], test panels [19], undersea cables [20], instrumented platforms on the seabed [21], etc. The factors influencing the distribution of sessile assemblages were identified as the light penetration, temperature, pressure, food and nutrient levels, nature of substratum, duration of immersion, and biology, physiology and dispersal of individual species [19,22].The density of sessile photosynthetic algae in the assemblages is determined by the decay of light levels in the water column, species-specific adaptations [23] and grazing[24]. Invertebrate taxa dominate the sessile assemblages below the photic zone and depend upon exogenous organic material for nutrition[22]. The exogenous organic material for invertebrate taxa in the seabed and seamounts are transported by currents and other physical processes driven by the topographical features[25–27]. The influence of large topographical features such as seamounts in altering the environment and increased availability of organic matter in the benthic nepheloid layers[28]plays an important role in the establishment of sessile assemblages in an otherwise hostile condition.But, the man-made stationary structures such as moored buoys and petroleum platforms are much smaller and unlikely to alter the physical environment of the adjacent water column. Consequently, the sessile assemblages on such structures are believed to be dictated by the environmental parameters typical to the pelagic ocean [13,15].

**Fig 1:**
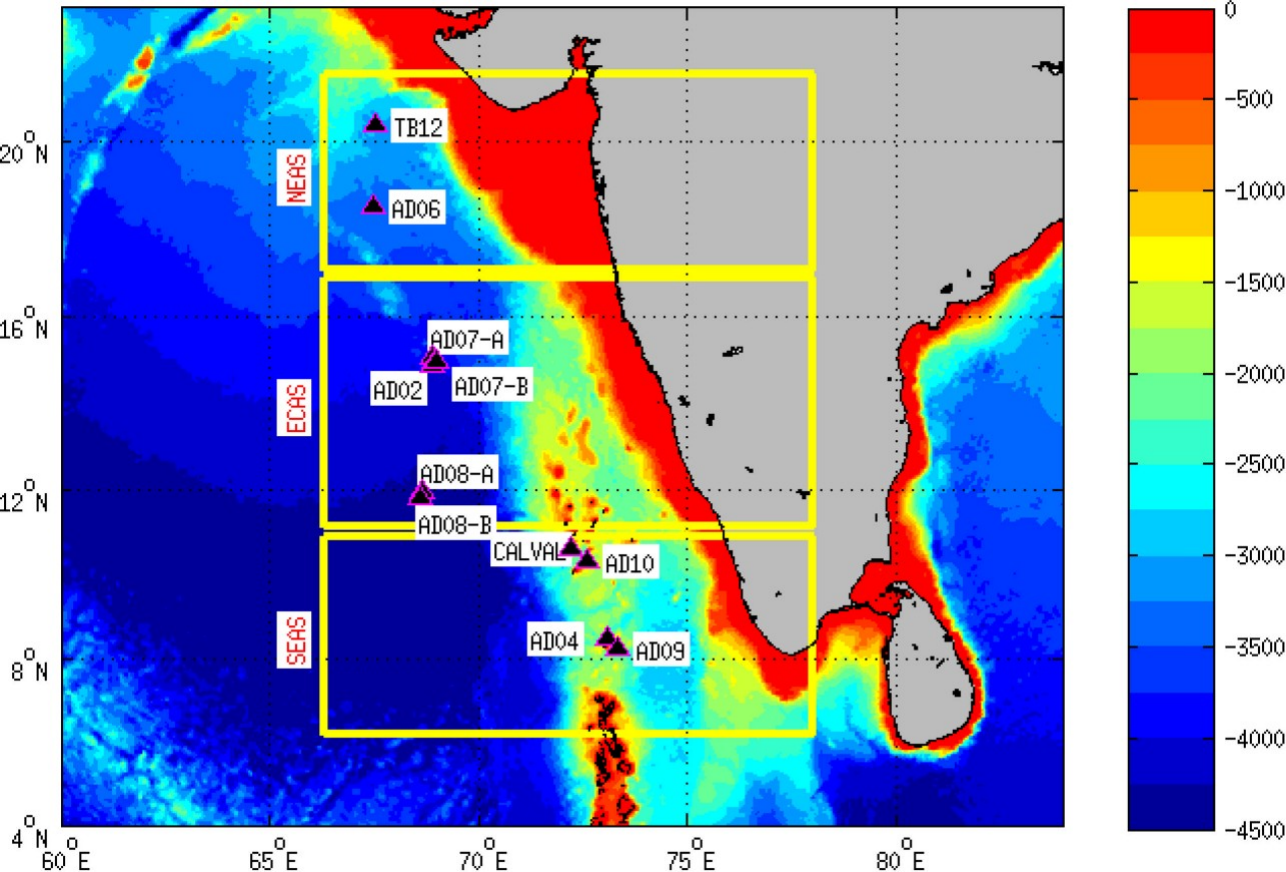
Depth of seabed (in meters)in the eastern Arabian Sea (Arabian Sea east of 65°E) and the surrounding oceanic regions based on ETOPO2v2 data. Yellow boxes indicate the three sub-regions, namely southeastern Arabian Sea (SEAS, 6–11°N), east-central Arabian Sea (ECAS, 11–17°N) and northeastern Arabian Sea (NEAS, 17–23°N). Markers on the map indicate the locations of moored buoys.

Large-scale spatial patterns of assemblages in the marine environment are primarily driven by abiotic stress [7,29–32]. In this study, we have analyzed whether the patterns of macrofoulant assemblages on a network of moored buoys could potentially be explained by spatiotemporal variability of environmental parameters. Eleven moored buoys deployed in an area covering ~1×10^6^ km^2^ in the eastern Arabian Sea (Fig 1) were selected for the study. The Arabian Sea is a tropical ocean basin that exhibits pronounced seasonality in key environmental parameters. In the subsequent sections, we have documented the patterns of macrofoulant assemblages on the moored buoys and analyzed the influence of environmental parameters.

## Data and methods

### Measuring geographic distribution of macrofoulants

Patterns of macrofoulants were observed on moorings deployed in the eastern Arabian Sea for a durationof 128 to 477 days (Fig 1 and Table 1).The moorings examinedduring the study comprised of four configurations, namely Met-Ocean buoy (AD02 and AD04), CALVAL, tsunami buoy (TB12) and Ocean Moored buoy for North Indian Ocean (OMNI, moorings AD09, AD10, AD08-A, AD08-B, AD07-A, AD07-B and AD06). Moorings AD08-A and AD08-B were located only 16 km apart and hence are collectively referred as the AD08 in subsequent text. Similarly, AD07-A and AD07-B are collectively referred as AD07 as they were located only 14 km apart. Field permit for the research was granted by the National Institute of Ocean Technology, Chennai, India.

**Table 1:** Details of moored data buoys sampled in the study.

Location ID/Region		Latitude	Longitude	Distance from nearest coast (km)	depth (m)	Deployment date	No. of days Deployed
SEAS	AD09	08.253°N	73.350°E	33	2250	04-07-2015	477
	AD04	08.491°N	73.096°E	23	2300	29-03-2016	208
	AD10	10.318°N	72.589°E	28	1800	31-08-2015	421
	CALVAL	10.605°N	72.230°E	26	2100	26-11-2015	335
ECAS	AD08-B	11.768°N	68.597°E	338	4320	23-06-2016	128
	AD08-A	11.910°N	68.636°E	338	4300	24-11-2015	340
	AD02	14.865°N	68.914°E	497	4030	03-09-2015	424
	AD07-B	14.966°N	68.994°E	497	3960	26-06-2016	128
	AD07-A	15.042°N	68.889°E	497	3996	19-11-2015	348
NEAS	AD06	18.515°N	67.471°E	397	3300	21-11-2015	349
	TB12	20.339°N	67.547°E	247	3030	14-11-2015	358

Distribution of macrofoulants on mooring was documented during retrieval of the system using photographic and scrape sampling techniques. Photographs of random quadrat measurements (0.25 x 0.25 m) were taken of all mooring components shallower than 5 m to estimate density of various fouling communities. Scrapes were made upto mooring substratum from all accessible mooring components. Individual organisms were sorted, preserved in 95% ethanol and identified to species level for the dominant fouling barnacle and to family or order for the other macrofoulants. Retrieval procedure of OMNI buoys permitted sampling from all depths and analysis of depth wise distribution of macrofoulants. For other type of moorings, samples from the components up to a depth of about four meters were collected, as rest of the mooring components were not retrieved. Total wet biomass on Conductivity-Temperature (CT) sensors deployed on OMNI buoys was estimated by subtracting the weight after retrieval from thatof a clean sensor. Zooplankton samples in the upper 100 mof the water column were collected by single vertical haul of zooplankton net (mesh size 300μm) near the moorings during OctoberandNovember 2016.

### Environmental data

Data on environmental parameters in the mooring locations were documented based on mooring mounted sensors, shipborne measurements during retrieval of the moorings, remote sensing products and other climatology datasets. SEA-BIRD CTsensors (SBE 37-SMP, SBE 37-IM) mounted on OMNI moorings at discrete depth levels 0.5, 1, 5, 10, 15, 20, 30, 50, 75, 100, 200 and 500 meters recorded temperature and salinity at hourly interval for entire deployment duration of the moorings.Profiles of temperature, salinity, chlorophyll-*a* and DO in the top 500 m in the vicinity of the mooring were measured using SBE 19plus V2 SeaCAT Profiler CTD during retrieval of the moorings. The *in-situ* datasets are archived by Indian National Centre for Ocean Information Services. Spatial distribution of salinity was studied based on World Ocean Atlas 2013 (WOA2013) [33]. Monthly climatology of remotely sensed surface chlorophyll-*a* concentration over the eastern Arabian Sea was used as a proxy for phytoplankton abundance.The chlorophyll-*a* data is based on the European Space Agency’s Climate Change Initiative (OC-CCIv3) mission [34]. Gridded monthly climatology of ocean surface current data used in the analysis was based on Ocean Surface Current Analysis-Real time (OSCAR), which is an estimate of mean currents in top 30 m of the ocean[35].The bathymetry data wasGridded Global Relief Data, Earth topography two arc-minute (ETOPO2) v2.

### Data analysis

Temporal variations in temperature and salinity at different depths in OMNI buoy locations were analyzed based on the hourly dataset collected by the moorings.Summary statistics of the temperature and salinity data were calculated to describe mean conditions, short-term variability and seasonal-scale variability. All statistics presented here are based on quality-controlled data during the period mentioned in Table 1. The average value of salinity and temperature at different depths in the mooring locations were obtained as the mean of the dataset during the entire deployment period. Diurnal variability of salinity and temperature were studied using a proxy called mean daily peak-to-peak variability. The mean daily peak-to-peak variability in salinity and temperature was computed as the mean of the difference between daily maximum and minimum.The standard deviation of salinityand temperature timeseries after smoothing out high-frequency variability using a three-day running mean is considered as a proxy to study seasonal scale variability. Higher values for the standard deviation are indicative of large seasonal variations in salinityand temperature.Linear correlations of the summary statistics with density distribution of the most predominant macrofoulant at different depths on the moorings were determined using Pearson correlation. All statistical analysis was performed in MATLAB R2019a.

## Results and discussion

### Distribution of foulants on moored buoys in the eastern Arabian Sea

Mooring components in the top 100 m of the water column were found to be colonized by diverse macrofouling organisms. On the basis of wet biomass, macrofoulant taxa on moorings were composed of 93–97% pedunculate barnacles, 1–3% algae, 1–2% bivalves, 1–2% hydroids and less than 1% other fouling organisms like bryozoans, sessile filter feeder polychaetes, non-sessile mobile organisms such as crab species, predatory polychaetes and echinoderms, etc. (Table 2).

**Table 2:** Macrofoulants observed on moorings and the zooplankton groups collected from vicinity of the moorings. Zooplankton samples were collected only from selected stations for moorings located less than 30 nautical miles apart from one another.

Mooring location, ID& Region	Macrofoulants observed on mooring components		Zooplankton groups collected from top 100 m of water column in vicinity of the mooring		
	Identified up to Order	Identified up to Family	Identified up to Order	Identified up to Family	Larval forms
08.25°N, 73.35°E AD09 [SEAS]	Pedunculata, Euphausiacea, Decapoda, Sabellida, Phyllodocida, Chlorophyceae*, Mytilida, Arcida, Ophiacanthida, Ostreida, Desmodorida	Lepadidae, Plagusiidae, Serpulidae, Nereididae, Mytilidae, Ophiocomidae, Amphiuridae, Pteriidae,	Amphioxiformes, Foraminiferida, Euphausiacea, Aphragmophora, Myodocopida	Acartiidae, Paracalanidae, Calanidae, Candaciidae, Lucicutiidae, Eucalanidae, Pontellidae, Oithonidae, Sapphirinidae, Corycaeidae, Euterpinidae	Copepod nauplii, Crab Zoea, Decapod larvae, Lepadidae cyprid, Fish eggs and larvae, Polychaete larvae
08.49°N, 73.10°E AD04 [SEAS]	Pedunculata, Sabellida, Decapoda, Euphausiacea, Ostreida, Chlorophyceae*, Monhysterida	Lepadidae, Serpulidae, Plagusiidae,Pteriidae, Linhomoeidae	Data not available		
10.32°N, 72.59°E AD10 [SEAS]	Pedunculata, Euphausiacea, Decapoda, Sabellida, Ostreida,Ophiacanthida, Amphinomida, Phyllodocida, Chlorophyceae*	Lepadidae, Plagusiidae, Serpulidae,Pteriidae, Nereididae, Amphinomidae, Ophiocomidae	Aphragmophora, Myodocopida, Euphausiacea	Acartiidae, Paracalanidae, Calanidae, Lucicutiidae, Eucalanidae, Pontellidae, Timoridae, Oithonidae, Oncaeidae, Corycaeidae	Decapod larvae, Cyphonautes larvae, Crab zoea, Copepod nauplii, Lepadidae cyprid
10.61°N, 72.23°E CALVAL [SEAS]	Pedunculata, Decapoda, Euphausiacea, Ostreida, Chlorophyceae*	Lepadidae, Plagusiidae, Pteriidae,	Data not available		
11.77°N, 68.60°E AD08-B [ECAS]	Pedunculata, Lepadiformes, Decapoda, Euphausiacea, Phyllodocida, Chlorophyceae*	Lepadidae, Poecilasmatidae, Plagusiidae, Nereididae	Data not available		
11.91°N, 68.64°E AD08-A [ECAS]	Pedunculata, Lepadiformes, Phyllodocida, Ostreida, Euphausiacea, Decapoda, Cheilostomata, Haplotaxida, Monhysterida, Chlorophyceae*	Lepadidae, Poecilasmatidae, Nereididae,Pteriidae, Plagusiidae, Membraniporidae, Xyalidae, Polygordiidae	Lepadiformes, Foraminiferida, Euphausiacea, Aphragmophora, Myodocopida	Luciferidae, Paracalanidae, Clausocalanidae, Calanidae, Pseudocalanidae, Eucalanidae, Candaciidae, Oithonidae, Oncaeidae, Corycaeidae, Oikopleuridae	Copepod nauplii, Lepadidae cyprid, Lepadidae nauplii, Poecilasmatidae nauplii, *Conchoderma*sp. cyprid, Crab zoea, Polychaete larvae
14.86°N, 68.91°E AD02 [ECAS]	Pedunculata, Decapoda, Euphausiacea, Ostreida, Phyllodocida, Chlorophyceae*	Lepadidae, Plagusiidae, Pteriidae, Nereididae	Data not available		
14.96°N, 68.99°E AD07-B	Pedunculata, Lepadiformes, Decapoda, Euphausiacea, Phyllodocida, Chlorophyceae*	Lepadidae, Poecilasmatidae, Plagusiidae, Nereididae	Data not available		
15.04°N, 68.88°E AD07-A [ECAS]	Pedunculata, Lepadiformes, Decapoda, Euphausiacea, Mytiloida, Enterogona, Phyllodocida, Ostreida, Amphinomida, Oscillatoriales, Chlorophyceae*	Lepadidae, Poecilasmatidae, Plagusiidae, Mytilidae, Clavelinidae, Nereididae, Pteriidae, Amphinomidae, Phormidiaceae	Aphragmophora, Euphausiacea	Clausocalanidae, Calanidae, Candaciidae, Pontellidae, Oithonidae, Oncaeidae, Corycaeidae, Oikopleuridae	Bivalve veliger, Lepadidae cyprid, Lepadidaenauplii, Poecilasmatidae nauplii, Copepod nauplii, Polychaete larvae
18.51°N, 67.47°E AD06 [ECAS]	Pedunculata, Lepadiformes, Decapoda, Euphausiacea, Phyllodocida, Chlorophyceae*, Copelata, Gelidiales, Oscillatoriales	Lepadidae, Poecilasmatidae, Plagusiidae, Nereididae, Pteriidae, Microcoleaceae, Oikopleuridae	Foraminiferida, Euphausiacea, Aphragmophora, Myodocopida	Acanthoniidae, Podonidae, Clausocalanidae, Calanidae, Candaciidae, Diphyidae, Lucicutiidae, Pontellidae, Oithonidae, Oncaeidae, Sapphirinidae, Corycaeidae, Oikopleuridae	Polychaete larvae, Lepadidae nauplii, Poecilasmatidae nauplii, Copepod nauplii,
20.34°N, 67.55°E TB12 [ECAS]	Pedunculata, Euphausiacea, Decapoda, Sabellida,Chlorophyceae*, Ostreida,Enterogona	Lepadidae, Plagusiidae,Pteriidae, Serpulidae, Clavelinidae	Euphausiacea, Aphragmophora,Myodocopida	Podonidae, Luciferidae, Paracalanidae, Calanidae, Metridinidae, Lucicutiidae, Pseudocalanidae, Eucalanidae, Pontellidae, Oithonidae, Oncaeidae, Sapphirinidae, Corycaeidae	Lepadidae cyprid, Fish larvae, Copepod nauplii,

*Identified up to Class.

**Fig 2:**
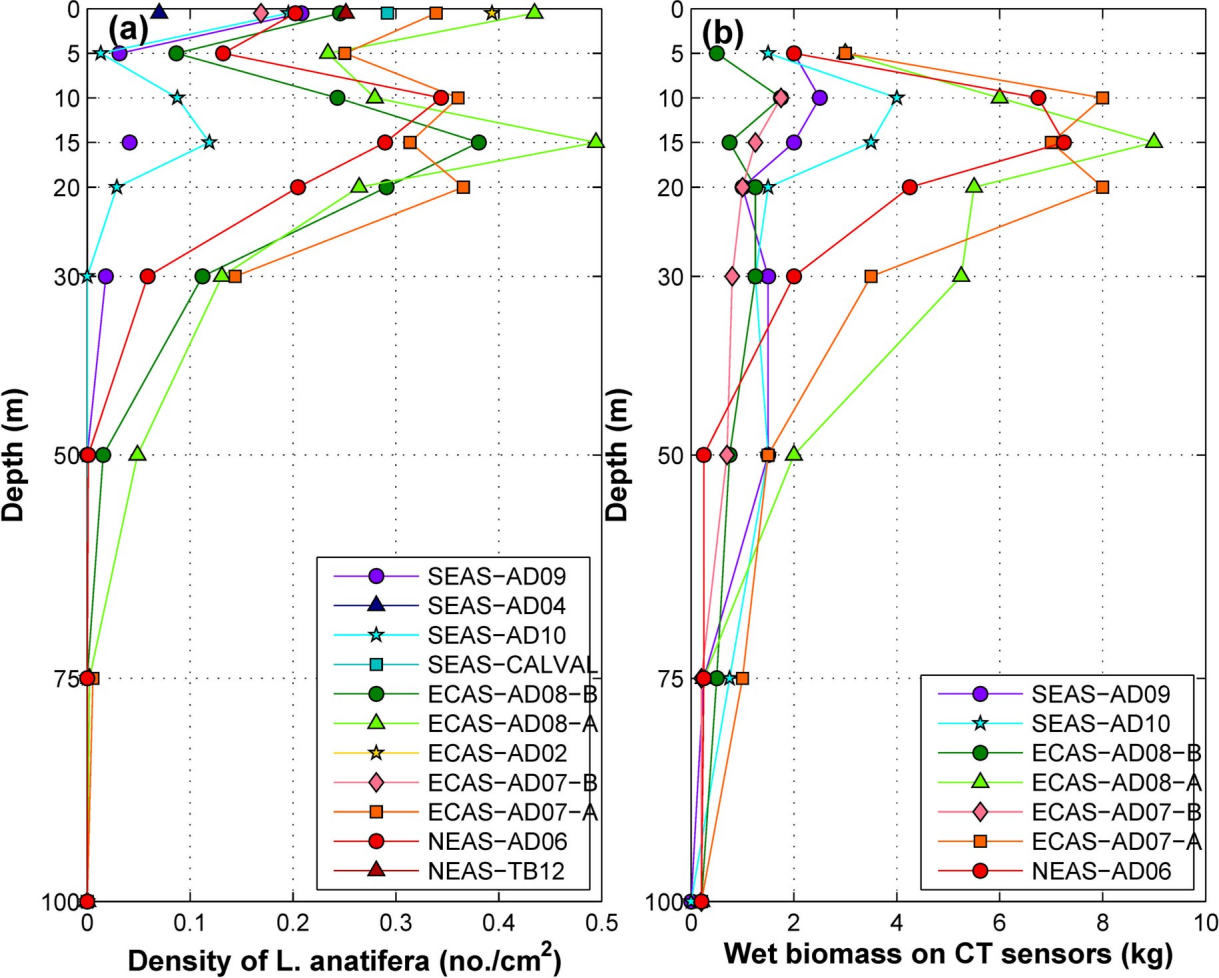
(a) Density of *L*. *anatifera* (no./cm^2^) on buoy and Conductivity-Temperature (CT) sensors of moorings in the Eastern Arabian Sea (b) Wet biomass (kg) of foulants on CT sensors retrieved from different depth levels at OMNI mooring locations.

The semi-submerged buoys of all eleven moorings were nearly identical (S1–S3 Figs). Fouling was observed to be concentrated around the rubber fender fixed at the waterline and on the ridges of the corners. Mean density of the most predominant macrofoulant *Lepasanatifera* on fender and edges of surface buoy of moorings are given in Fig 2A (corresponding to depth 0.5 m). In the southeastern Arabian Sea (SEAS), density of *L*. *anatifera*was observed to be very low (0.2±0.09no./cm^2^) on surface buoys (Fig 2A and S1 Table). Buoys deployed for more than 420 days in the SEAS (S1B and S1D Fig) had only low to moderate fouling. The moorings deployed in the east-central Arabian Sea (ECAS) for more than 340 days namely, AD08-A, AD07-A and AD02 were the most fouled (S2B, S2C, S2E and S2F Fig) with high density of *L*. *anatifera* (0.4±0.06no./cm^2^). Buoys deployed in the ECAS for a shorter duration of ~130 days, namely, AD08-B (S2D Fig) and AD07-B (S2A Fig) were also colonized by juvenile *L*. *anatifera* with a density of 0.22±0.06 no./cm^2^. Moderate levels of *L*. *anatifera* (0.23±0.04 no./cm^2^, Fig 2A) were observed on surface buoys of moorings in the northeastern Arabian Sea (NEAS) (S3 Fig).

**Fig 3:**
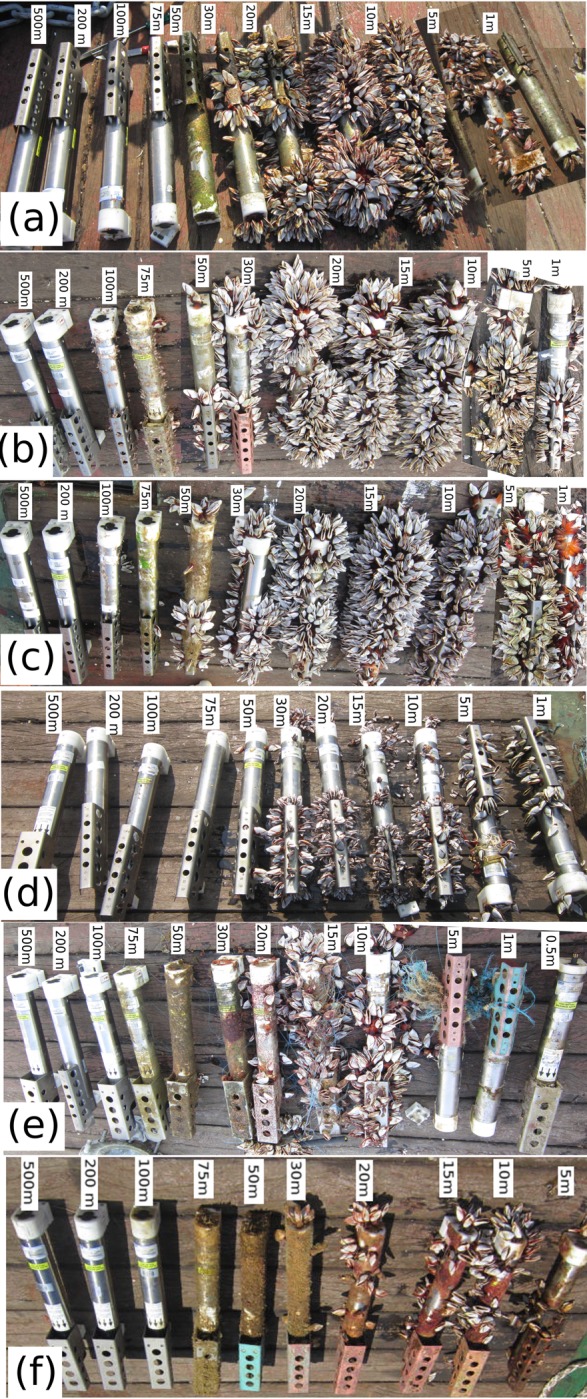
Conductivity-Temperature sensors retrieved from OMNI buoys (a) NEAS-AD06, (b) ECAS-AD07, (c) ECAS-AD08-A, (d) ECAS-AD08-B, (e) SEAS-AD10 and (f) SEAS-AD09. The labels in the figure indicate depth at which the CT sensors were deployed.

The vertical distribution of fouling organisms colonizing the buoy and its mooring system were investigatedbased on data from OMNI buoys. The algal communities were observed on all moorings in the depths from 0 to 75 m. The dominant macrofoulant species colonizing subsurface mooring components were pedunculate barnacles, hydroids, polychaetes, oysters, etc. The density and wet biomass of the macrofoulants varied with depth. The Macrofoulants were noticeable up to a depth of 75 m in the SEAS, 130 m for the ECAS and 120 m for the NEAS. The pedunculate barnacle, *L*. *anatifera* was dominant from 0 to 50m (Fig 2). Besides, a few specimens of *L*. *anatifera* were observed even at 75m depth on moorings in the ECAS and NEAS.At 75 to 130 m depth, the moorings in the ECAS and NEAS were mainly colonized by pedunculate barnacles *Conchodermahunteri* and *Octolasmiswarwickii*, and their maximum density was observed around 100m depth.The analysis of vertical profiles of wet biomass on all moorings revealed subsurface maxima in the depth range 10 to 20 m (Fig 2B), which coincided with high barnacle abundance (Figs 2A and 3). Consequently, the vertical profile of foulant wet biomass was nearly identical to the vertical distribution of *L*. *anatifera* (Fig 3). The analysis in S1 Appendix suggests that the distribution of macrofoulant assemblages documented herein were not significantly affected by the factors like dissimilarities in deployment duration, antifouling measures and retrieval procedures among moorings.

### Environmental control on spatial variability of fouling communities

Macrofoulant assemblages on eleven moored buoys retrieved from the open ocean waters of the Arabian Sea revealed the existence of distinct regional patterns. Ecological and oceanographic factors might explain the observed distributions. Herein, the possible influences of various ecological and oceanographic factors in governing the regional patterns were investigated.

#### Biological productivity

**Fig 4:**
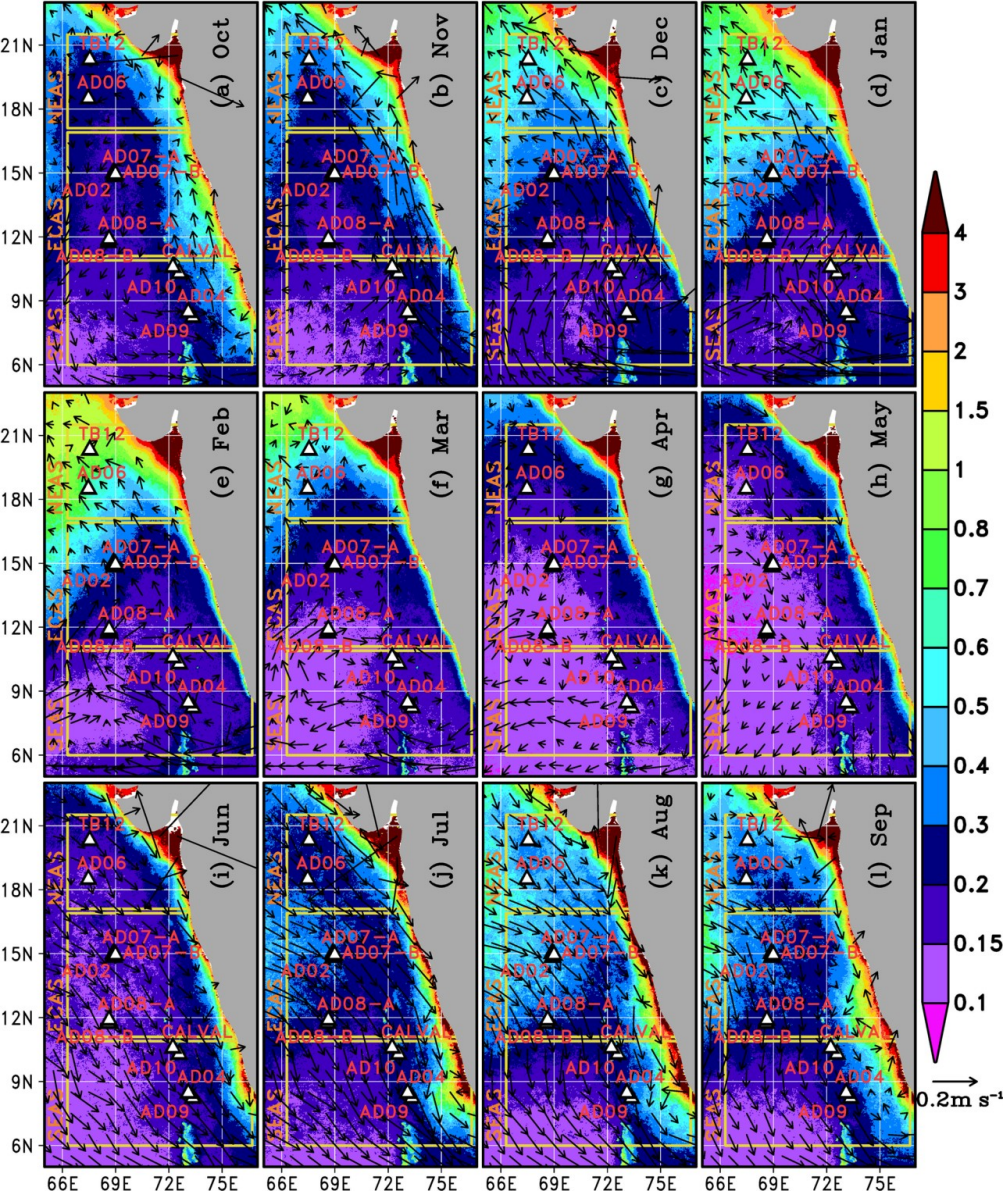
Color shading shows monthly climatology of chlorophyll-*a* (mg m^-3^) in the eastern Arabian Sea based on OC-CCIv3 data. Vectors show monthly climatology of surface currents based on the OSCAR data. Triangular markers indicate the locations of the moored buoys.

**Fig 5:**
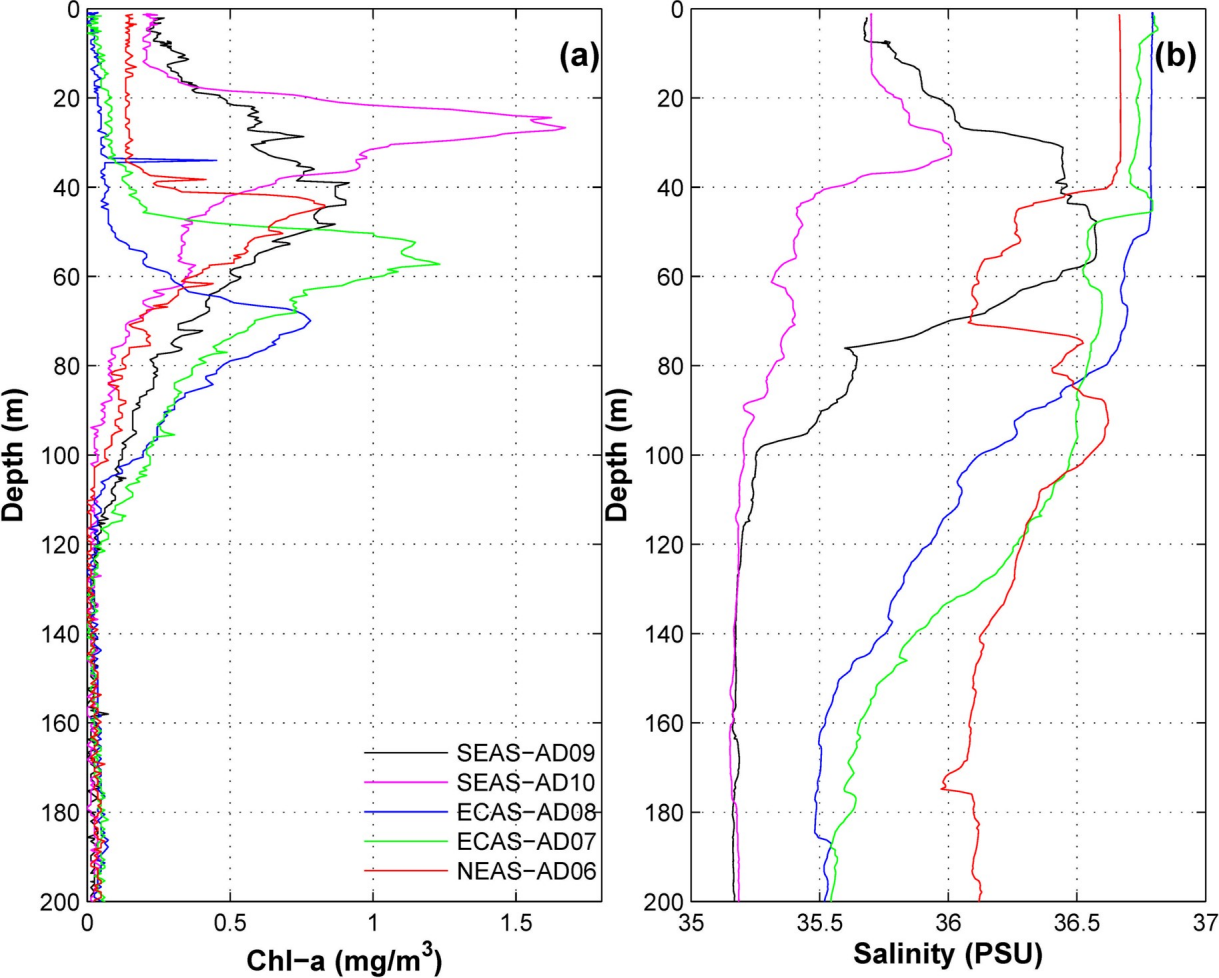
Vertical profiles of (a) chlorophyll-*a* (mg m^-3^) and (b) salinity (PSU) based on CTD casts during October-November 2016.

Several studies have reported increased growth of organisms in various trophic levels, aided by the upwelling driven availability of nutrients, phytoplankton and detritus [29,36–40]. We used remotely sensed and in situ chlorophyll-a data (Figs 4 and 5A) as proxies for phytoplankton abundance. Remote sensing gives an average value for chlorophyll-a in the top 25 meters[41]. High concentration of chlorophyll-*a*was observed in the NEAS (Fig 4A–4L). But, the chlorophyll-*a* concentration was perennially low in the ECAS. Moderately high chlorophyll-*a* concentration was observed in the vicinity of SEAS moorings during July to October. Vertical profile of chlorophyll-*a* in upper 200mof water column based on CTD casts in the vicinity of moorings (Fig 5A) was also consistent with the monthly climatology data (Fig 4A–4L).Relatively low concentration of chlorophyll-*a* observed in the ECAS in comparison to SEAS and NEAS suggests that ECAS was least productive region. However, the moorings in the least productive area within the study region had the highest density of macrofoulants (Figs 2 and 3). Hence, it is apparent that factors other than biological productivity also contribute significantly to the spatial variability of macrofoulant wet biomasson moorings. Previous studies suggest that pelagic production constitutes the dominant basal resource fueling sessile suspension-feeding organisms on artificial reefs[24].The role of changes in concentration of planktonic food in determining the vertical distribution of filter feeders on the mooring has to be further examined.

#### Zooplankton community analysis

The zooplankton samples were collected during the mooring retrieval. The density of zooplankton was higher in NEAS (~5000 no./m^3^) in comparison to ECAS and SEAS (~2000 no./m^3^)(S4 Fig). Copepods were the dominant taxain all zooplankton sampling locations but they did not account for the fouling. Among the fouling community, nauplii and cyprids of the stalked barnacle, *L*. *anatifera*was observed in most of the stations (Table 2). ECAS and NEAS also recorded the presence of Poecilasmatidae nauplii. Zooplankton samples from all stations lacked larval forms of acorn barnacles, substantiating the absence of adult acorn barnacles from the moorings. The absence of acorn barnacles could be attributed to the offshore location of the stations[42–44]. Although Mytilidae and Ostreidae were observed on moorings, the zooplankton community lacked bivalve larvae, except for bivalve veliger in ECAS. The absence of bivalve larvae could be attributed to seasonality of reproduction exhibited by the species.

Most of the biofouling taxa on mooring components, viz. barnacles, bivalves, bryozoans, polychaetes, hydrozoans etc. have a planktonic larval stage in their life cycle[45]. However, the fouling community is not a replica of the zooplankton community. The variations between biofoulants and zooplankton communities could be due to the dispersal capability of larval forms, which are susceptible to competition, predation, mortality and advection by ocean currents[46](Fig 4). Besides, the zooplankton distributions reported here are based on point samples of larvae from organisms which live for months to years.

#### Salinity

**Fig 6:**
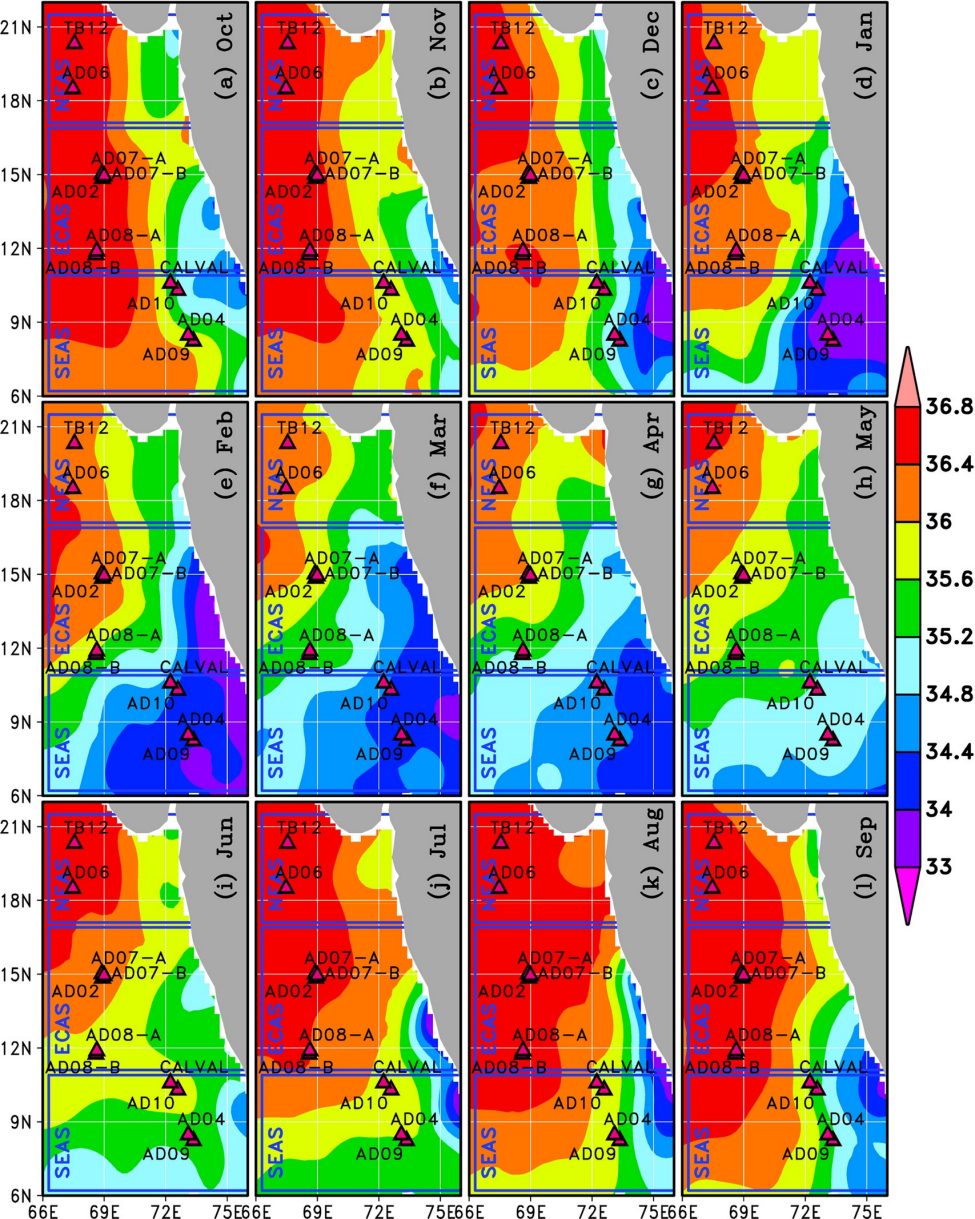
Monthly climatology of sea surface salinity (in PSU) in the eastern Arabian Sea based on WOA2013 [33]. Triangular markers indicate the locations of the moored buoys.

**Fig 7:**
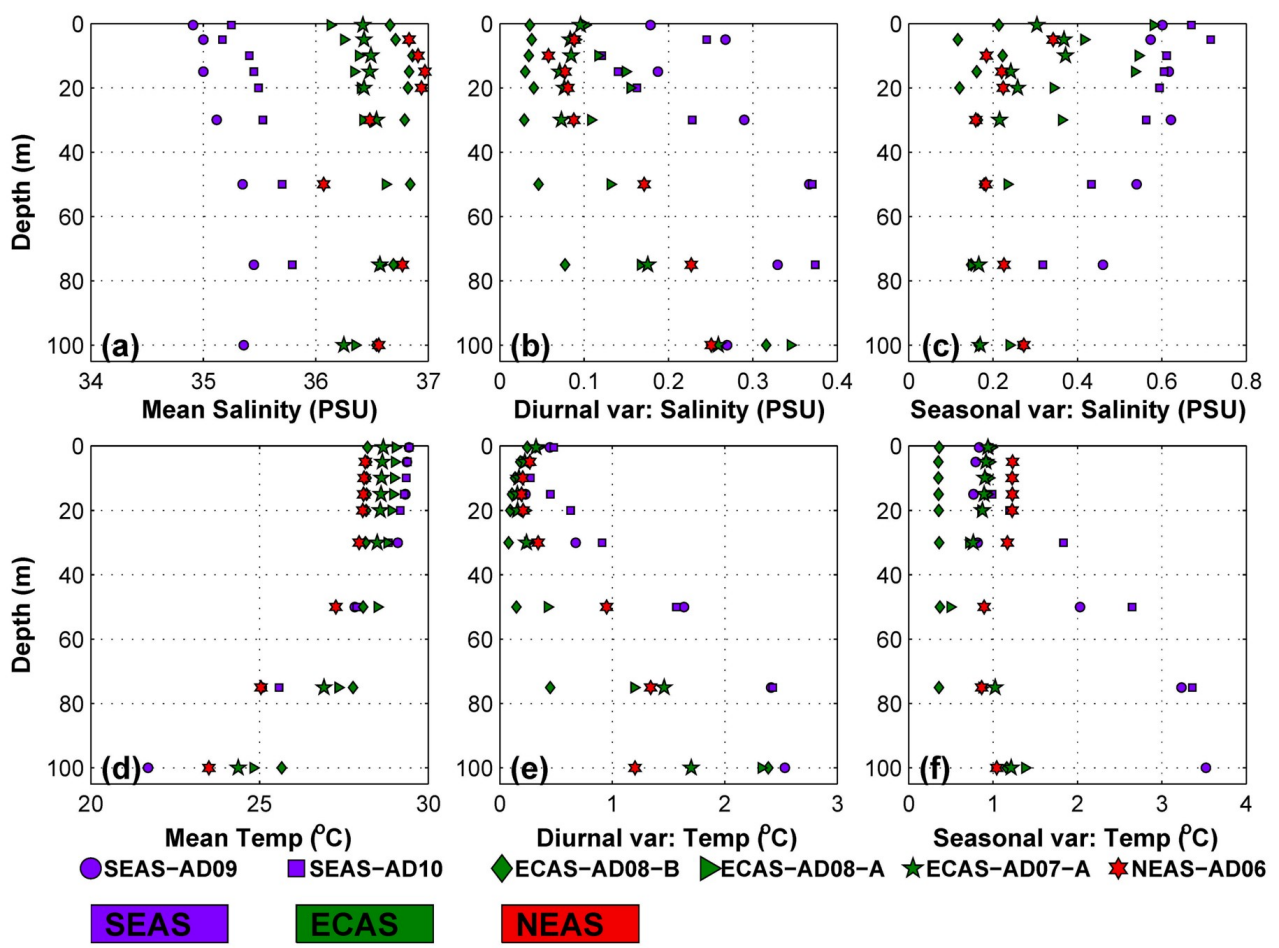
Scatter plot showing depth versus (a) mean salinity, (b) mean daily peak-to-peak variability in salinity, (c) standard deviation of salinity after smoothing the time series using 3-day running mean, (d) mean temperature, (e) mean daily peak-to-peak variability in temperature and (f) standard deviation of temperature after smoothing the time series using 3-day running mean. Different marker types are used to identify moorings. Keys for the marker types and marker colors are given at the bottom of the figure.

The eastern Arabian Sea is part of a highly saline tropical ocean [47]. Seasonally reversing monsoon currents in the north Indian Ocean [48]advect low-saline water from the Bay of Bengal into the SEAS during the period from November to April. Consequently, salinity in the SEAS rapidly decreases during November to April (Fig 6B–6G). Vertical profile of salinity based on CTD casts (Fig 5B) also suggests that salinity in upper 200min theSEASwas lower than that of ECAS and NEAS. Fig 7A–7C showcertain summary statistics of temporal evolution of salinity in top 100 m of water column at the mooring locations. Mean salinity in the mooring locations were in the range of 35 to37 PSU (Fig 7A). The mean salinity in the SEAS was about 1 PSU lower than that of ECAS and NEAS (Fig 7A). On average, daily variations in salinity, determined as mean daily peak-to-peak variability in salinity, was less than 0.4 PSU in the eastern Arabian Sea, with highest daily variations recorded in the SEAS and lowest in the ECAS (Fig 7B). Salinity in the SEAS mooring locations showed large seasonal variations as evident from the high standard deviation of salinity (Fig 7C). The large seasonal scale salinity variations in the SEAS were a consequence of changes in circulation patterns (Fig 4A–4L and Fig 5A–5L).

**Fig 8:**
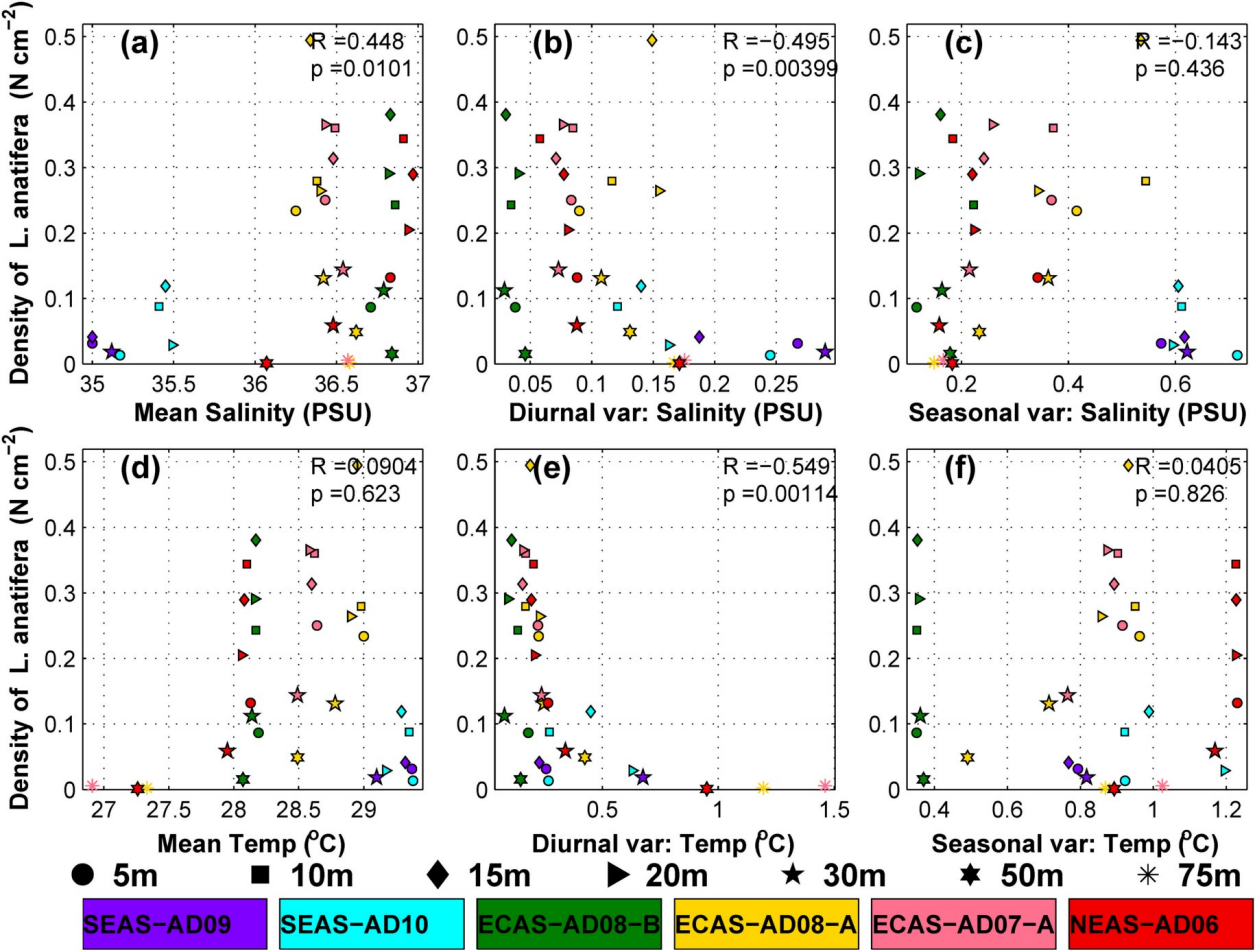
Scatter plot showing abundance of *L*. *anatifera* (no./cm^2^) versus (a) mean salinity, (b) mean daily peak-to-peak variability in salinity, (c) standard deviation of salinity after smoothing the timeseries using 3-day running mean, (d) mean temperature, (e) mean daily peak-to-peak variability in temperature and (f) standard deviation of temperature after smoothing the time series using 3-day running mean. Different marker types are used to identify the depth levels in water column. The markers are also color coded to distinguish respective mooring locations. Keys for the marker types and marker colors are given at the bottom of the figure.

Fig 8A–8C shows scatter plot of density of *L*. *anatifera* on CT sensors in the depth range 5 to 75 m versus different statistics of salinity. The density of *L*. *anatifera* showed a significant positive correlation (R = 0.45, p = 0.011) with mean salinity. The significant positive correlation suggests that density of *L*. *anatifera* could increase with increase in mean salinity. Significant negative correlation (R = -0.495, p = 0.004) was observed between the mean daily peak-to-peak variability in salinity and density of *L*. *anatifera*(Fig 8B). Seasonal variability of salinity did not show any significant correlation with density of *L*. *anatifera* (Fig 8C) in the eastern Arabian Sea.Hence, it is apparent that the prevailing low-saline conditions and strong diurnal variability of salinity contributedsignificantly to the lowdensity of *L*. *anatifera* on moorings in the SEAS.

#### Temperature

Temperature plays a vital role in regulating respiration, feeding and breeding of marine organisms [49,50]. Tolerance of marine organisms to ambient temperature showed marked variations across species [51,52]. Recruitment of larvae on substrates were also influenced by temperature [53–55]. Hence, temperature could potentially regulate species composition and vertical zonation of macrofoulants on moorings. The most predominant macrofoulant on the mooring, *L*. *anatifera* is usually found in tropical oceans having temperature >18°C. Laboratory experiments suggests that *L*. *anatifera* could reproduce in temperatures ranging from 15 to 30°C, with 19 to 27°C being the most optimal breeding temperature [56].

**Fig 9:**
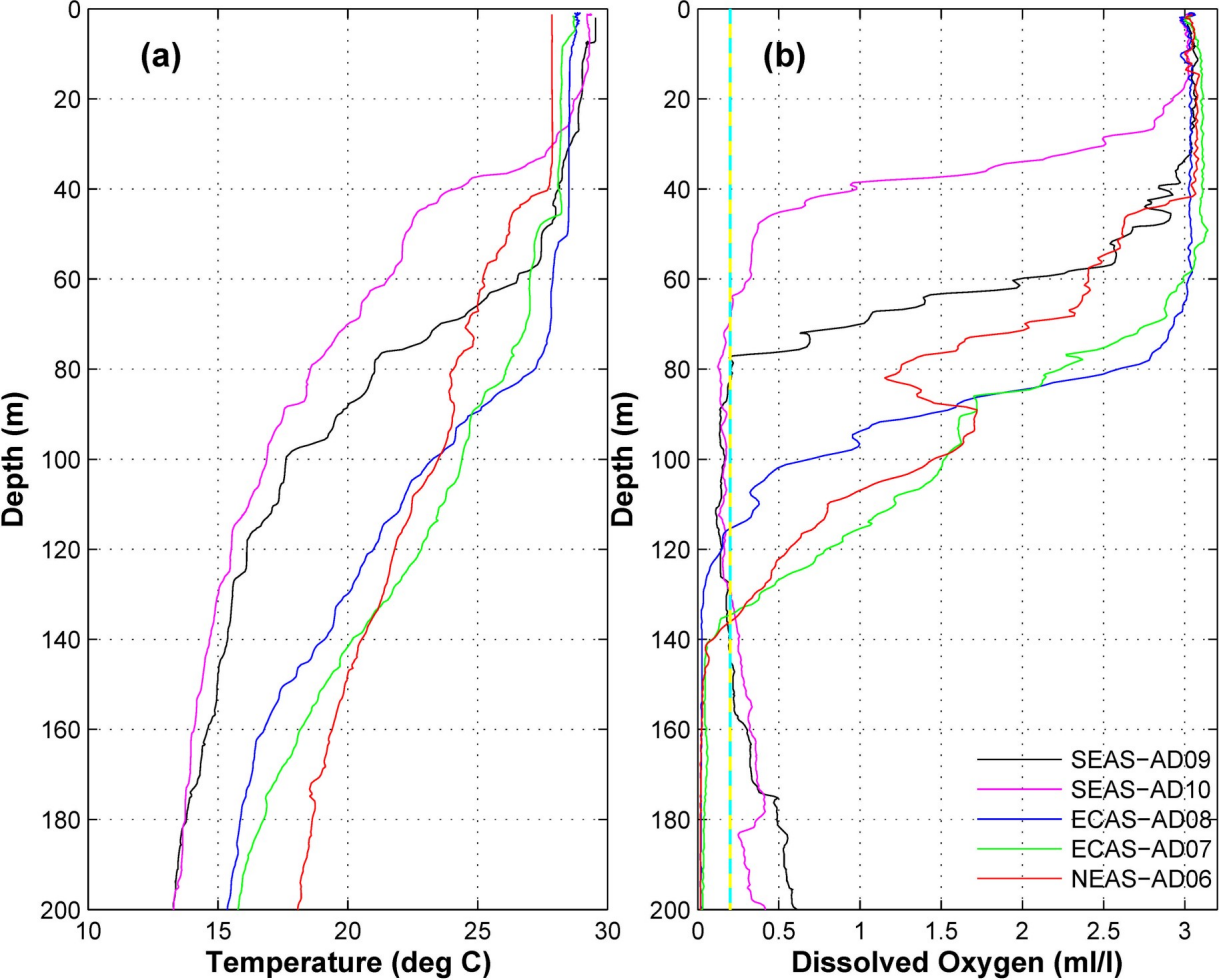
Vertical profiles of (a) temperature (°C) and (b) DO (ml l^-1^) based on CTD casts during October-November 2016. The CTD casts were performed in the vicinity of OMNI buoys before retrieval of the moorings. The dashed vertical line in (b) indicates DO level of 0.2 ml l^-1^, which is hypoxic and physiologically stressful for marine organisms[62].

Different statistics of temperature evolution in the eastern Arabian Sea were analyzed to study the influence of temperature on biofouling. Vertical profile of temperature based on mean data during the mooring deployment period (Fig 7D) and CTD casts during October 2016 (Fig 9A) revealed a warm mixed layerhaving minimal variation of temperature with depth. A significant fraction of the biofoulants observed on the mooring was concentrated withinthis warm layer in top 50 m (Fig 3). Also, majority of *L*. *anatifera* observed on moorings were in the water column having mean temperature in the range of 28 to 29.5°C(Fig 8D). But density of *L*. *anatifera* exhibited insignificant correlation with mean temperature (Fig 8D), apparently due to the nearly identical mean temperature values throughout the eastern Arabian Sea (Fig 7D). Previous studies have shown that diurnal temperature fluctuations can influence community structure in marine environments [57,58]. Diurnal temperature variability in the eastern Arabian Sea was studied based on mean daily peak-to-peak variability in temperature (Fig 7E). In all mooring locations, average daily variations in temperature was only about 0.5°C at depth 15 to 20 m, while much larger daily temperature variations were observed in other depths of the water column.The larger values of mean daily peak-to-peak variability in temperature in the water column deeper than 20 m was due to internal tides having diurnal and semidiurnal periodicities[59,60]. High frequency temperature variability was most pronounced in the SEAS moorings (Fig 7E). Temperature data close to sea surface also had diurnal and semidiurnal oscillations (Fig 7E). The high-frequency temperature variability penetrating up to a depth of 10 m from the sea surface was driven by factors such as turbulence, air-sea fluxes and diurnal solar heating [61]. In the eastern Arabian Sea moorings, *L*. *anatifera* was observed in the water column that recorded mean daily peak-to-peak variability of temperature less than 1.5°C (Fig 8E). Significant negative correlation (R = -0.549, p = 0.001) was observed between density of *L*. *anatifera* and mean daily peak-to-peak variability in temperature (Fig 8E). Hence, it is plausible that relatively stable temperature in water column in the depth range 15 to 20 m could have promoted increased growth of *L*. *anatifera*, leading to the formation of subsurface maxima (Fig 2).Standard deviation of temperature time series after smoothing out high frequency variability using a three-day running mean (Fig 7F) is considered as a proxy to study seasonal scale variability of temperature. Relatively higher standard deviations of temperature were observed in the water column deeper than 20m in the SEAS pointing to the large seasonal variability of temperature in the region compared to both NEAS and ECAS. But, density of *L*. *anatifera*did not show any significant correlation with seasonal variability of temperature (Fig 8F).

#### Dissolved oxygen

Availability of DO in the marine environment is a crucial factor that regulates metabolic and biogeochemical processes. The near-surface layer of the ocean is replete with DO. But, the subsurface depths has a much lower concentration of DO, wherein oxygen advected from surface layer is consumed by respiration, decay of sinking organic matter etc.[63]. The depleted level of DO in water depth ranging from 10 to 1300 m with a concentration less than 0.5 ml l^-1^ is called mid-depth Oxygen Minimum Zone (OMZ) [64]. The Arabian Sea with its closed northern boundary encompasses the second-most intense OMZ of all tropical oceans [62].

Profiles of DO in the vicinity of moored buoys based on CTD casts are plotted in Fig 9B. The concentration of DO in the near-surface layer was ~3 ml l^-1^ at all mooring locations. The thickness of the well-mixed oxygen-repletenear-surface layer was observed to be highest in the ECAS (~65 m) and lowest in the SEAS (~30m) (Fig 9B). DO depleted rapidly in oxycline located below near-surface layer at all mooring locations. DO in the NEAS and ECAS decreased to microxic levels (<0.1 ml l^-1^, [65])at ~140mdepth. But in the SEAS, DO first decreased to hypoxic levels (<0.2 ml l^-1^) at ~70mand then increased with depth from 140m(Fig 9B). The higher concentrationof DO in intermediate depths in the SEASwas driven by advection of DO from southern hemisphere [66,67]. The oxycline was very shallow and had strong vertical gradientin the SEAS(Fig 9), which indicates prevalence of strong upwelling in the region.Time series*in-situ* measurement of DO in the SEAS also revealed the occurrence of pronounced seasonality associated with upwelling [68].Macrofoulants with limited or no motility may be exposed to physiological stress associated with episodes of hypoxia. Seasonal occurrence of hypoxia could devastate marine environment [69–71]. Hence, the varying levels of oxygen stress can be instrumental in shaping composition and vertical zonation of macrofoulants on moorings. Hypoxic conditions in the SEAS at a shallow depth of 75 m could be a significant contributor for the absence of macrofoulants below 75 m on moorings.

The deeper near-surface DO replete water columnin the ECAS (Fig 9B) had*L*. *anatifera* assemblages up to 75 m depth.*C*. *hunteri* and *O*. *warwickii* were predominant macrofoulants in the depth range 75 to 130 m on moorings in the NEAS and ECAS. In these regions, the depth range 75 to 130 m coincided with oxycline and thermocline wherein both temperature and DO decreased rapidly with depth (Fig 9A and 9B). The prevalence of strong vertical gradients could regularly cause huge fluctuations in temperature and DO as the water column oscillates due to internal tides. Thus it is apparent that the *C*. *hunteri* and *O*. *warwickii* thriving in depth 75 to 130 m on moorings in the NEAS and ECAS were better adapted to physiological stress associated with depleted DO and semidiurnal oscillations in DO and temperature.

## Conclusion

Macrofoulant assemblages on moored buoys spread across an area of ~1×10^6^ km^2^ in the eastern Arabian Sea exhibited significant spatial variability in terms of species composition, vertical zonation and wet biomass. Our analysis revealed that the observed patterns of macrofoulant assemblages were shaped by various environmental parameters. Biological productivity and zooplankton community distribution in the vicinity of the moorings were observed to have only a limited influence in shaping the macrofoulant assemblages.

A correlation analysis based on temperature and salinity suggests that the most predominant foulant on moorings,*L*. *anatifera* was less tolerant to low-saline environments and diurnal variations in both temperature and salinity. But, the mean temperature and seasonal variability in temperature and salinity prevalent in the near-surface waters of the eastern Arabian Sea had only a limited influence on *L*. *anatifera* abundance. The results also suggested that the higher density of *L*. *anatifera* in the ECAS was aided by the relatively stable near-surfacetemperature and salinity conditions prevalent in this region.

The impact of artificial substratum, foraging and other biotic factors on the macrofouling were not considered in the present study. Previous studies in shallow coastal environments revealed that assemblages on artificial substrates were not identical to that of adjacent natural substrates as they offer atypical surfaces in terms of orientation, depth range and surface [72,73]. Foraging also plays an important role in shaping the assemblages on artificial structures [74]. The influence of these factors in determining the mesoscale patterns of macrofoulant assemblages on artificial structures have to be further investigated.

Broad scale warming observed in the global ocean [75–77] as well as interannual warming or cooling events such as El Niño–Southern Oscillation (ENSO) could induce significant impact on marine ecosystems [78]. Ourstudy suggests that the impact of anomalies in ocean temperature or salinity on marine ecosystems could be reflected in the abundance of *L*. *anatifera* on artificial structures in the marine environment. Previous studies have shown that certain epifaunal taxa of foulant communities found on the mooring components such as foraminifera, nematoda, polychaeta, etc. are good bioindicators of ocean acidification [79], ocean warming [80] and hypoxic environments [64,81]. Many of the common macrofoulants on moorings are also considered as good bioindicators for anthropogenic impacts [82].These organisms could, potentially act as bioindicators to monitor changes in the pelagic ecosystems, which constitute about 99 percent of earth’s biosphere[83]. Moored buoys are distributed in all major oceanic regionsas part of Global Tropical Moored Buoy Array as well as other regional networks enabling real-time *in-situ* ocean observation[84]. Global distribution of moored buoys, coupled with the availability of high-quality scientific data about its environment make them an ideal platform to monitor the effect of environment on macrofoulant assemblages. Sustained monitoring of mesoscale patterns of macrofoulant assemblages on moored buoys could significantly enhance our understanding of changes in pelagic ecosystem associated with the global changes in climate.

## Supporting information

S1 Appendix Potential impact of dissimilar deployment duration, antifouling measures and retrieval procedures of moored buoys on observed spatial patterns of macrofoulant assemblage. (DOCX)

S1 Table Density of *L*. *anatifera* on mooring components at different depth levels. The density at depth 0.5 m is based on the quadrat measurements on buoy hull and the density at all other depths are based on the count of *L*. *anatifera* on CT sensors at designated depths. Different statistics based on hourly time series measurement of temperature and salinity from sensors mounted on mooring corresponding to entire deployment duration is also given.
(DOCX)

S2 Table Wet biomass (in kg) of macrofoulants observed on CT sensors deployed at designated depth levels on OMNI moorings. (DOCX)

S1 Dataset Vertical profiles of temperature, salinity, chlorophyll-a, dissolved oxygen, turbidity and pH based on CTD casts in the eastern Arabian Sea during October-November 2016. (XLS)

S1 Fig Biofouling on surface buoys of moorings in the southeastern Arabian Sea (a) CALVALdeployed for335 days at 11°N, 72°E, (b) AD10 deployed for421 days at 10°N, 73°E, (c) AD04 deployed for208 days at 9°N, 73°E and (d) AD09deployed for 477 days at 8°N, 73°E.
(TIF)

S2 Fig Biofouling on surface buoys of moorings in the east-central Arabian Sea (a) AD07-Bdeployed for128 days at 15°N, 69°E, (b) AD07-Adeployed for348 days at 15°N, 69°E, (c) AD02deployed for424 days at 15°N, 69°E, (e) AD08-Bdeployed for128 days at 12°N, 69°E, (e and f) AD08-Adeployed for 340 days at 12°N, 69°E.
(TIF)

S3 Fig Biofouling on surface buoys of moorings in the northeastern Arabian Sea (a and b) AD06 deployed for349 days at 19°N, 68°E and (c) TB12deployed for358 days at 20°N, 67°E.
(TIF)

S4 Fig Zooplankton abundance in the mooring stations in top 100 m of water column. (TIF)
