# Supplementary material for: Influence of environmental factors on macrofoulant assemblages on moored buoys in the eastern Arabian Sea
Source: PLoS One. 2020 Jan 30;15(1):e0223560. doi: 10.1371/journal.pone.0223560 (PMC6992173; doi:10.1371/journal.pone.0223560)
Supplement: S1 Appendix — (DOCX) [file pone.0223560.s001.docx]

S1 Appendix**: Potential impact of dissimilar deployment duration, antifouling measures and retrieval procedures of moored buoys on observed spatial patterns of macrofoulant assemblage**

The eleven moorings analyzed in this study differed from one another with respect to mooring type, retrieval procedure, deployment duration and antifouling measures. It is imperative to scrutinize whether these factors introduced any significant biases in observed spatial patterns of biofouling. Deployment duration of moorings was non-uniform (Table 1). Nevertheless, five out of eleven moorings analyzed here were distributed in three local regions and deployed for a nearly identical duration of 335 to 358 days (Fig 1, Table 1). Clear regional patterns of macrofoulant assemblages could be observed in the subset of five moorings having nearly identical deployment duration. Similarly, moorings AD10 and AD02 deployed for about 420 days in the southeastern and east-central Arabian Sea respectively accumulated distinct levels of macrofoulant growth (S1b Fig and S2c Fig). Hence, it can be argued that the inferences drawn on regional patterns of macrofoulant assemblages might not be significantly affected by the dissimilar deployment duration of moorings.

Retrieval procedure of the OMNI moorings permitted the analysis of the depth wise distribution of biofouling communities in the water column. But four moorings, AD02, AD04, CALVAL and TB12, were of different design and their retrieval procedure did not permit the documentation of biofoulants deeper than four meters in the water column. Nevertheless, except for TB12, the other three moorings were located near the OMNI moorings (Fig 1) wherein a detailed sampling was performed. Hence, the insufficiency in sampling of the four mooring locations may not cause any significant bias in the observations.

Few of the CT sensors on moorings analyzed in this study had copper on it as an antifouling measure. Guards made of copper were used instead of stainless steel in those CT sensors on an experimental basis. Copper guards were used on CT sensors at all depth levels shallower than 50 meters on AD09 (Fig 2f), at 1 and 5 meters in AD10 (Fig 2e) and 30 meters on AD07-A (Fig 2b). No or negligible biofouling was observed on copper guards on the retrieved CT sensors. However, it is apparent that the copper guards did not induce any significant reduction in biofouling on other components of the CT sensor (Fig 2). Remarkably, a majority of the CT sensors that employed copper guards were deployed on moorings AD09 and AD10 in the southeastern Arabian Sea, where biofouling was observed to be minimal. Copper guards fixed on few CT sensors on the mooring were unlikely to limit biofouling on other mooring components. Relatively low biofouling observed on surface buoys of moorings AD09 and AD10 as well as that of AD04 and CALVAL suggests that the low levels of biofouling was a consistent pattern in the southeastern Arabian Sea irrespective of antifouling measures used. Besides, biofouling on moorings retrieved from the locations of AD09 and AD10 on previous years were also analyzed based on archived photographs (not shown). The analysis reveals that biofouling on moorings retrieved earlier from AD09 and AD10 locations were nearly identical to those analyzed here even when copper guards were not used. The evidence suggests that the low levels of biofouling observed in the southeastern region of Arabian Sea may not be an artifact arising from the uneven use of antifouling measures.
