# Supplementary material for: Influence of environmental factors on macrofoulant assemblages on moored buoys in the eastern Arabian Sea
Source: PLoS One. 2020 Jan 30;15(1):e0223560. doi: 10.1371/journal.pone.0223560 (PMC6992173; doi:10.1371/journal.pone.0223560)
Supplement: S1 Table — The density at depth 0.5 m is based on the quadrat measurements on buoy hull and the density at all other depths are based on the count of L. anatifera on CT sensors at designated depths. Different statistics based on hourly time series measurement of temperature and salinity from sensors mounted on mooring corresponding to entire deployment duration is also given. (DOCX) [file pone.0223560.s002.docx]

**S1 Table**: Density of *L. anatifera* on mooring components at different depth levels. The density at depth 0.5 m is based on the quadrat measurements on buoy hull and the density at all other depths are based on the count of *L. anatifera* on CT sensors at designated depths. Different statistics based on hourly time series measurement of temperature and salinity from sensors mounted on mooring corresponding to entire deployment duration is also given.

| **Region, Buoy-ID and Depth** | | | **Density of *L. anatifera* (no./cm^2^)** | **Mean Temperature (Deg C)** | **Mean Salinity (PSU)** | **Mean daily Peak-to-peak variability in temperature (Deg C)** | **Mean daily Peak-to-peak variability in salinity (PSU)** | **Standard deviation of 3-Day mean temperature (Deg C)** | **Standard deviation of 3-Day mean salinity (PSU)** |
| --- | --- | --- | --- | --- | --- | --- | --- | --- | --- |
| SEAS | AD09 | 0.5m | 0.2083 | 29.43 | 34.91 | 0.4452 | 0.1787 | 0.8342 | 0.6012 |
|  |  | 5m | 0.0314 | 29.37 | 35.00 | 0.2579 | 0.2675 | 0.7935 | 0.5733 |
|  |  | 15m | 0.0413 | 29.32 | 35.00 | 0.2282 | 0.1875 | 0.7681 | 0.6168 |
|  |  | 30m | 0.0182 | 29.10 | 35.12 | 0.6761 | 0.2899 | 0.8175 | 0.6219 |
|  |  | 50m | 0.0000 | 27.82 | 35.35 | 1.6377 | 0.3668 | 2.0314 | 0.5405 |
|  |  | 75m | 0.0000 | 25.07 | 35.45 | 2.4133 | 0.3293 | 3.233 | 0.4602 |
|  |  | 100m | 0.0000 | 21.70 | 35.36 | 2.5344 | 0.2694 | 3.5213 | 0.2706 |
|  | AD04 | 0.5m | 0.0700 | 29.51 | 35.16 | 0.3116 | 0.124 | 1.1595 | 0.4713 |
|  | AD10 | 0.5m | 0.1955 | 29.44 | 35.25 | 0.4805 | 0.6152 | 0.9571 | 0.6702 |
|  |  | 5m | 0.0132 | 29.38 | 35.17 | 0.2685 | 0.2452 | 0.9231 | 0.7156 |
|  |  | 10m | 0.0877 | 29.35 | 35.41 | 0.272 | 0.1209 | 0.9218 | 0.6114 |
|  |  | 15m | 0.1188 | 29.29 | 35.45 | 0.4506 | 0.1402 | 0.9882 | 0.6053 |
|  |  | 20m | 0.0289 | 29.17 | 35.49 | 0.6294 | 0.1627 | 1.1949 | 0.5945 |
|  |  | 30m | 0.0000 | 28.82 | 35.53 | 0.9098 | 0.2281 | 1.8334 | 0.563 |
|  |  | 50m | 0.0000 | 27.88 | 35.70 | 1.5701 | 0.3703 | 2.6481 | 0.4332 |
|  |  | 75m | 0.0000 | 25.58 | 35.79 | 2.4257 | 0.374 | 3.3615 | 0.318 |
|  | CALVAL | 0.5m | 0.2917 | 29.41 | 35.48 | 0.3692 | 0.1058 | 1.0368 | 0.6945 |
| ECAS | AD08-B | 0.5m | 0.2457 | 28.20 | 36.66 | 0.2456 | 0.0356 | 0.36 | 0.2136 |
|  |  | 5m | 0.0866 | 28.19 | 36.71 | 0.1815 | 0.0378 | 0.3511 | 0.1163 |
|  |  | 10m | 0.2431 | 28.17 | 36.86 | 0.136 | 0.0344 | 0.3519 | 0.2227 |
|  |  | 15m | 0.3807 | 28.17 | 36.83 | 0.1092 | 0.0301 | 0.3533 | 0.1611 |
|  |  | 20m | 0.2909 | 28.16 | 36.82 | 0.0938 | 0.0403 | 0.3569 | 0.1203 |
|  |  | 30m | 0.1121 | 28.14 | 36.79 | 0.0782 | 0.029 | 0.3615 | 0.1637 |
|  |  | 50m | 0.0155 | 28.07 | 36.84 | 0.1484 | 0.046 | 0.3703 | 0.1789 |
|  |  | 75m | 0.0000 | 27.77 | 36.69 | 0.4476 | 0.0774 | 0.359 | 0.148 |
|  |  | 100m | 0.0000 | 25.66 | 36.54 | 2.387 | 0.3158 | 1.1626 | 0.1663 |
|  | AD08-A | 0.5m | 0.4347 | 29.03 | 36.13 | 0.3413 | 0.1013 | 0.9806 | 0.5801 |
|  |  | 5m | 0.2339 | 29.00 | 36.25 | 0.2258 | 0.0899 | 0.9623 | 0.4154 |
|  |  | 10m | 0.2795 | 28.98 | 36.38 | 0.1692 | 0.1164 | 0.9502 | 0.5444 |
|  |  | 15m | 0.4944 | 28.95 | 36.34 | 0.1892 | 0.1491 | 0.9319 | 0.5352 |
|  |  | 20m | 0.2643 | 28.90 | 36.40 | 0.2299 | 0.1547 | 0.8574 | 0.343 |
|  |  | 30m | 0.1310 | 28.78 | 36.42 | 0.2481 | 0.1078 | 0.7141 | 0.3622 |
|  |  | 50m | 0.0488 | 28.49 | 36.62 | 0.4251 | 0.1311 | 0.4911 | 0.2334 |
|  |  | 75m | 0.0022 | 27.33 | 36.58 | 1.1944 | 0.1668 | 0.8689 | 0.149 |
|  |  | 100m | 0.0000 | 24.81 | 36.35 | 2.324 | 0.3445 | 1.3779 | 0.2382 |
|  | AD02 | 0.5m | 0.3933 | 28.93 | 36.38 | 0.0811 | 0.3098 | 0.8977 | 0.2746 |
|  | AD07-B | 0.5m | 0.1690 | 27.97 | 36.72 | 0.2035 | 0.03 | 0.3819 | 0.107 |
|  | AD07-A | 0.5m | 0.3391 | 28.67 | 36.42 | 0.3228 | 0.0956 | 0.9381 | 0.3039 |
|  |  | 5m | 0.2504 | 28.64 | 36.43 | 0.2226 | 0.0834 | 0.9154 | 0.3683 |
|  |  | 10m | 0.3604 | 28.62 | 36.49 | 0.1696 | 0.0847 | 0.9029 | 0.3717 |
|  |  | 15m | 0.3136 | 28.60 | 36.48 | 0.156 | 0.071 | 0.8927 | 0.2422 |
|  |  | 20m | 0.3655 | 28.58 | 36.43 | 0.1582 | 0.0764 | 0.8736 | 0.2582 |
|  |  | 30m | 0.1439 | 28.49 | 36.54 | 0.2386 | 0.073 | 0.7656 | 0.2152 |
|  |  | 75m | 0.0056 | 26.91 | 36.57 | 1.4614 | 0.1755 | 1.025 | 0.1659 |
|  |  | 100m | 0.0000 | 24.37 | 36.25 | 1.7018 | 0.259 | 1.2141 | 0.1691 |
| NEAS | AD06 | 0.5m | 0.2024 | -- | -- | -- | -- | -- | -- |
|  |  | 5m | 0.1321 | 28.13 | 36.83 | 0.2665 | 0.0882 | 1.2309 | 0.3425 |
|  |  | 10m | 0.3440 | 28.10 | 36.91 | 0.2035 | 0.0578 | 1.2263 | 0.184 |
|  |  | 15m | 0.2896 | 28.08 | 36.97 | 0.1943 | 0.0774 | 1.2277 | 0.2205 |
|  |  | 20m | 0.2049 | 28.06 | 36.94 | 0.2083 | 0.0807 | 1.2263 | 0.2239 |
|  |  | 30m | 0.0588 | 27.95 | 36.48 | 0.3414 | 0.0879 | 1.1695 | 0.1589 |
|  |  | 50m | 0.0011 | 27.26 | 36.07 | 0.9504 | 0.1712 | 0.8931 | 0.1827 |
|  |  | 75m | 0.0000 | 25.04 | 36.77 | 1.3413 | 0.2271 | 0.8637 | 0.2256 |
|  |  | 100m | 0.0000 | 23.50 | 36.56 | 1.2027 | 0.2507 | 1.0431 | 0.2727 |
|  | TB12 | 0.5m | 0.2514 | -- | -- | -- | -- | -- | -- |
