# Supplementary material for: Influence of environmental factors on macrofoulant assemblages on moored buoys in the eastern Arabian Sea
Source: PLoS One. 2020 Jan 30;15(1):e0223560. doi: 10.1371/journal.pone.0223560 (PMC6992173; doi:10.1371/journal.pone.0223560)
Supplement: S2 Table — (DOCX) [file pone.0223560.s003.docx]

**S2 Table**: Wet biomass (in kg) of macrofoulants observed on CT sensors deployed at designated depth levels on OMNI moorings.

| Depth (m) | AD09 | AD10 | AD08-B | AD08-A | AD07-B | AD07-A | AD06 |
| --- | --- | --- | --- | --- | --- | --- | --- |
| 0.5 | 0.5 | -- | 0.25 | 0.25 | -- | 1 | 0.75 |
| 1 | 1 | 1.25 | 0.75 | 3 | 0.2 | 2 | 0.5 |
| 5 | 2 | 1.5 | 0.5 | 3 | -- | 3 | 2 |
| 10 | 2.5 | 4 | 1.75 | 6 | 1.75 | 8 | 6.75 |
| 15 | 2 | 3.5 | 0.75 | 9 | 1.25 | 7 | 7.25 |
| 20 | 1 | 1.5 | 1.25 | 5.5 | 1 | 8 | 4.25 |
| 30 | 1.5 | 1.25 | 1.25 | 5.25 | 0.8 | 3.5 | 2 |
| 50 | 1.5 | 1.5 | 0.75 | 2 | 0.7 | 1.5 | 0.25 |
| 75 | 0.25 | 0.75 | 0.5 | 0.25 | 0.2 | 1 | 0.25 |
| 100 | 0 | 0 | 0.2 | 0.2 | 0.2 | 0.2 | 0.2 |
| 200 | 0 | 0 | 0 | 0 | 0 | 0 | 0 |
| 500 | 0 | 0 | 0 | 0 | 0 | 0 | 0 |
