# Supplementary figures and images for: Influence of environmental factors on macrofoulant assemblages on moored buoys in the eastern Arabian Sea
Source: PLoS One. 2020 Jan 30;15(1):e0223560. doi: 10.1371/journal.pone.0223560 (PMC6992173; doi:10.1371/journal.pone.0223560)

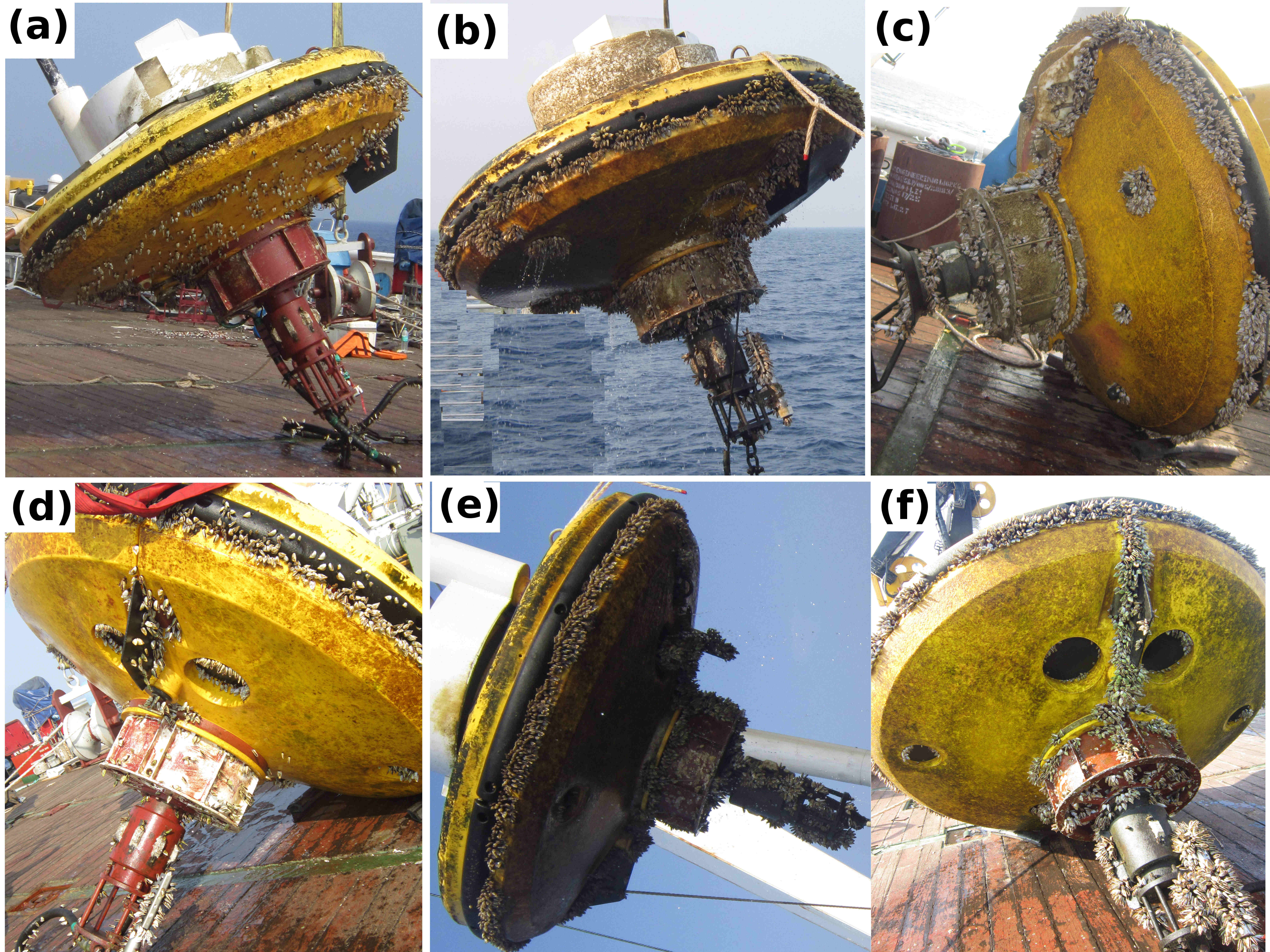

Supplement: S2 Fig — Biofouling on surface buoys of moorings in the east-central Arabian Sea (a) AD07-Bdeployed for128 days at 15°N, 69°E, (b) AD07-Adeployed for348 days at 15°N, 69°E, (c) AD02deployed for424 days at 15°N, 69°E, (e) AD08-Bdeployed for128 days at 12°N, 69°E, (e and f) AD08-Adeployed for 340 days at 12°N, 69°E. (TIF) [file pone.0223560.s006.tif]

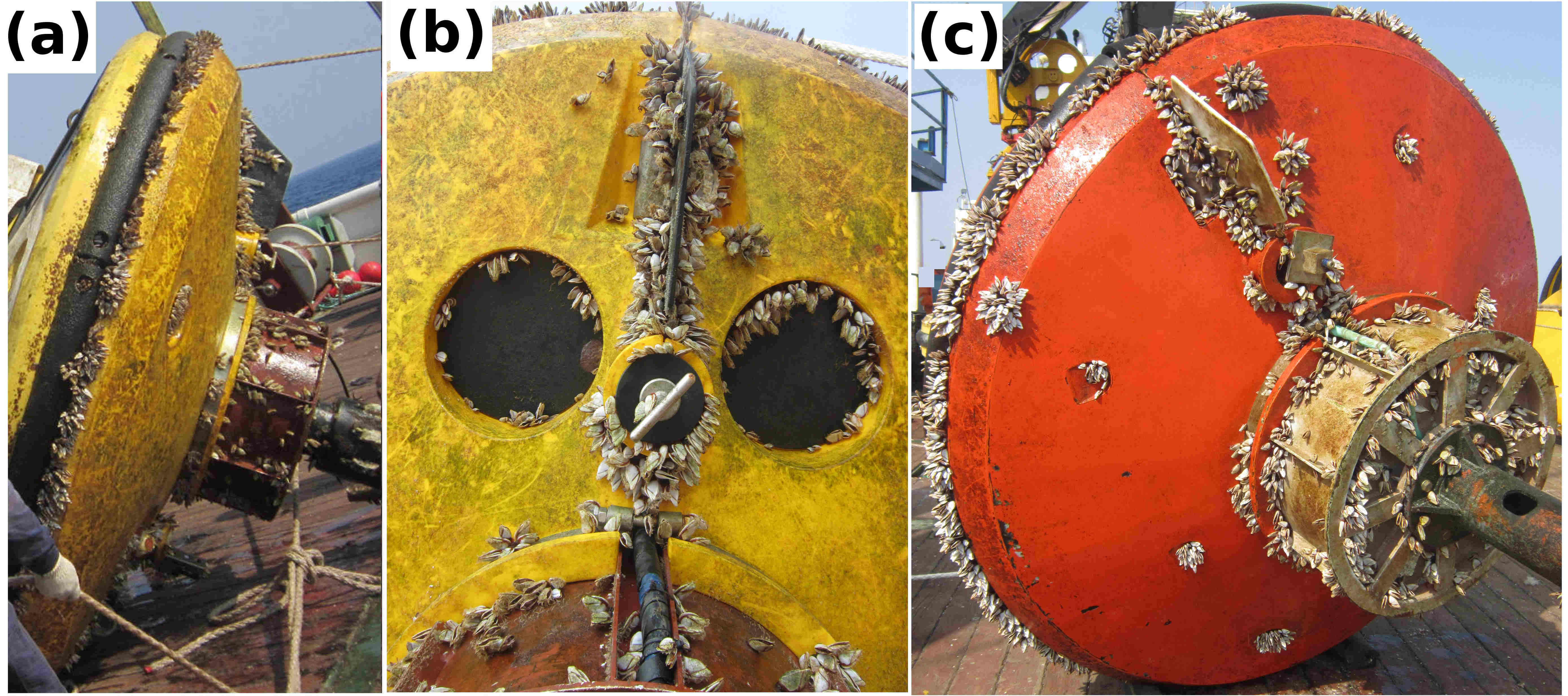

Supplement: S3 Fig — Biofouling on surface buoys of moorings in the northeastern Arabian Sea (a and b) AD06 deployed for349 days at 19°N, 68°E and (c) TB12deployed for358 days at 20°N, 67°E. (TIF) [file pone.0223560.s007.tif]

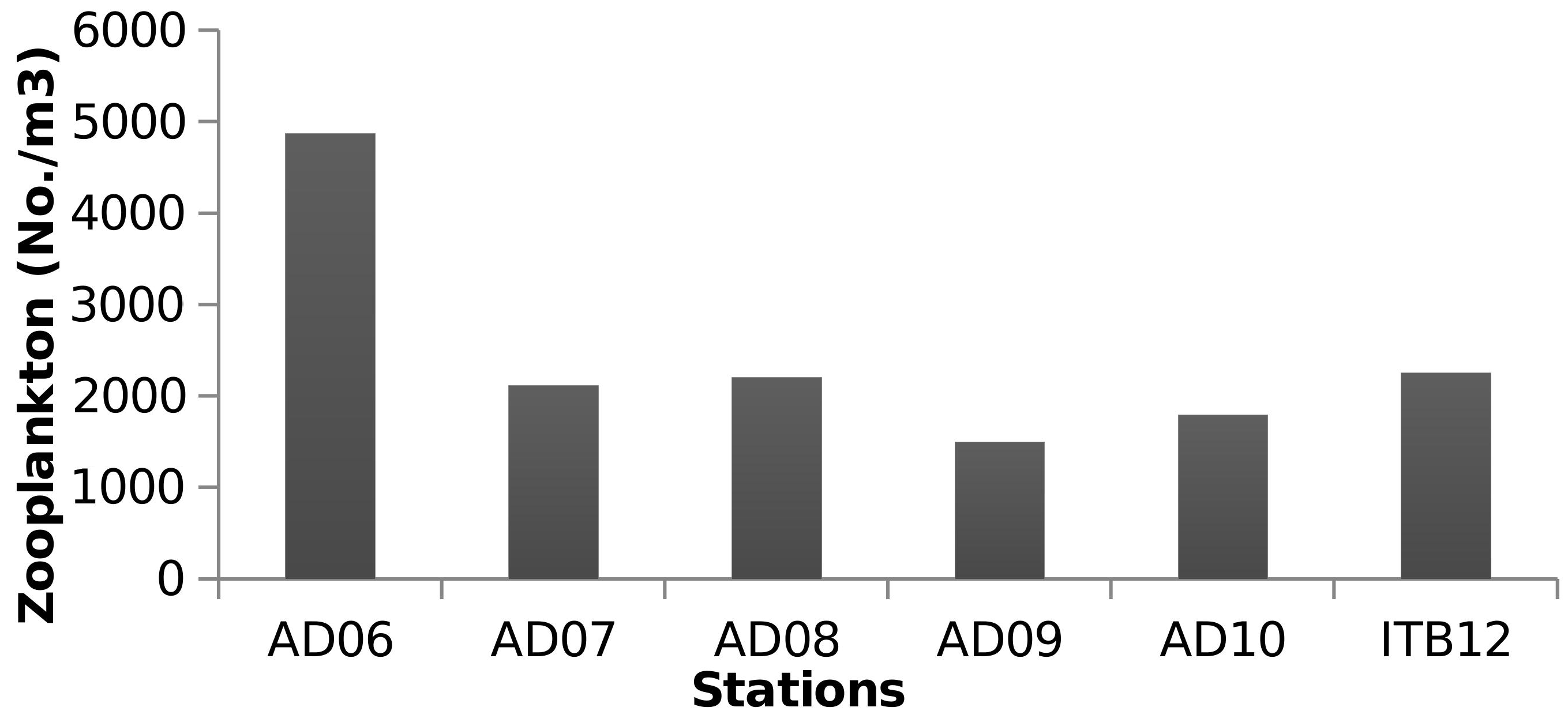

Supplement: S4 Fig — (TIF) [file pone.0223560.s008.tif]
